# GLP‐1 neurons form a local synaptic circuit within the rodent nucleus of the solitary tract

**DOI:** 10.1002/cne.24482

**Published:** 2018-09-19

**Authors:** J. Patrick Card, Aaron L. Johnson, Ida J. Llewellyn‐Smith, Huiyuan Zheng, Rishi Anand, Daniel I. Brierley, Stefan Trapp, Linda Rinaman

**Affiliations:** ^1^ Department of Neuroscience University of Pittsburgh Pittsburgh Pennsylvania; ^2^ Systems Neuroscience Center University of Pittsburgh Pittsburgh Pennsylvania; ^3^ Cardiovascular Medicine, Human Physiology and Centre for Neuroscience College of Medicine and Public Health, Flinders University Bedford Park South Australia Australia; ^4^ Department of Psychology Florida State University Tallahassee Florida; ^5^ Centre for Cardiovascular and Metabolic Neuroscience, Department of Neuroscience, Physiology & Pharmacology University College London London United Kingdom

**Keywords:** GABA, glutamate, local circuit network, noradrenergic, NTS, preproglucagon, prolactin‐releasing peptide, RRID: AB_2314562, RRID: AB_300798, RRID: AB_518978, synapse

## Abstract

Glutamatergic neurons that express pre‐proglucagon (PPG) and are immunopositive (+) for glucagon‐like peptide‐1 (i.e., GLP‐1+ neurons) are located within the caudal nucleus of the solitary tract (cNTS) and medullary reticular formation in rats and mice. GLP‐1 neurons give rise to an extensive central network in which GLP‐1 receptor (GLP‐1R) signaling suppresses food intake, attenuates rewarding, increases avoidance, and stimulates stress responses, partly via GLP‐1R signaling within the cNTS. In mice, noradrenergic (A2) cNTS neurons express GLP‐1R, whereas PPG neurons do not. In this study, confocal microscopy in rats confirmed that prolactin‐releasing peptide (PrRP)+ A2 neurons are closely apposed by GLP‐1+ axonal varicosities. Surprisingly, GLP‐1+ appositions were also observed on dendrites of PPG/GLP‐1+ neurons in both species, and electron microscopy in rats revealed that GLP‐1+ boutons form asymmetric synaptic contacts with GLP‐1+ dendrites. However, RNAscope confirmed that rat GLP‐1 neurons do not express GLP‐1R mRNA. Similarly, Ca^2+^ imaging of somatic and dendritic responses in mouse ex vivo slices confirmed that PPG neurons do not respond directly to GLP‐1, and a mouse crossbreeding strategy revealed that <1% of PPG neurons co‐express GLP‐1R. Collectively, these data suggest that GLP‐1R signaling pathways modulate the activity of PrRP+ A2 neurons, and also reveal a local “feed‐forward” synaptic network among GLP‐1 neurons that apparently does not use GLP‐1R signaling. This local GLP‐1 network may instead use glutamatergic signaling to facilitate dynamic and potentially selective recruitment of GLP‐1 neural populations that shape behavioral and physiological responses to internal and external challenges.

## 1 INTRODUCTION

Neurons synthesizing pre‐proglucagon (PPG), the protein precursor for glucagon‐like peptide‐1 (GLP‐1), reside within the caudal nucleus of the solitary tract (cNTS) and adjacent medullary reticular formation. Apart from a small population of olfactory bulb interneurons that express PPG (Merchenthaler, Lane, & Shughrue, 1999; Thiebaud et al., 2016), neurons within the cNTS and medullary reticular formation appear to provide the sole source of synaptic GLP‐1 signaling within the central nervous system (CNS) (Cork et al., 2015; Gu et al., 2013; Larsen, Tang‐Christensen, Holst, & Orskov, 1997; Llewellyn‐Smith, Gnanamanickam, Reimann, Gribble, & Trapp, 2013; Merchenthaler et al., 1999; Rinaman, 1999b; Trapp & Cork, 2015). Research using rats and mice has established that central GLP‐1 signaling pathways contribute importantly to physiological and behavioral processes linked to inhibition of food intake, exploratory and approach behaviors, and reinforcement/reward, coupled with escalation of physiological and behavioral stress responses, including avoidance and anxiety‐like behaviors (Lachey et al., 2005; Maniscalco & Rinaman, 2018; Rinaman, 1999a, 2010a, 2010b; Trapp & Cork, 2015). These functions are achieved through an extensive GLP‐1 axonal projection system targeting GLP‐1 receptor (GLP‐1R)‐expressing neurons distributed throughout the forebrain, brainstem, and spinal cord (Cork et al., 2015; Gu et al., 2013; Llewellyn‐Smith et al., 2015; Merchenthaler et al., 1999; Rinaman, 2010; Trapp & Cork, 2015; Trapp & Richards, 2013). For example, GLP‐1+ axon terminals synapse onto hypophysiotropic neurons within the paraventricular nucleus of the hypothalamus (Sarkar, Fekete, Legradi, & Lechan, 2003), where GLP‐1R is expressed (Cork et al., 2015), and hypothalamic GLP‐1 activates the endocrine hypothalamic‐pituitary‐adrenal (HPA) stress axis in a GLP‐1R‐dependent manner (Kinzig et al., 2003).

GLP‐1R also is expressed within the cNTS in rats and mice (Cork et al., 2015; Merchenthaler et al., 1999; Reiner et al., 2016), where GLP‐1 may act locally to mediate or support at least some of the behavioral and physiological effects attributed to central GLP‐1 signaling pathways (Hayes, Bradley, & Grill, 2009; Hayes, Skibicka, & Grill, 2008; Richard et al., 2015; Skibicka, 2013). Indeed, intra‐NTS administration of GLP‐1R agonists in rats reduces food intake and operant responding for food reward, and also reduces place preference conditioned by palatable food access (Hayes et al., 2011; Kanoski, Rupprecht, Fortin, De Jonghe, & Hayes, 2012); conversely, food intake is increased in rats after local NTS blockade of GLP‐1Rs (Hayes et al., 2009). The local circuitry mediating these pharmacological effects is unclear. In mice genetically engineered to express yellow fluorescent protein (YFP) under control of the glucagon promoter, light microscopy revealed YFP+ fibers and varicosities (presumably arising from GLP‐1 neurons) in close apposition with catecholaminergic NTS neurons, including those that comprise the A2 noradrenergic cell group (Llewellyn‐Smith et al., 2013; Richard et al., 2015). Further, at least a subset of catecholaminergic NTS neurons express mRNA for GLP‐1R in mice (Cork et al., 2015). While similar localization studies have not been conducted in rats, central (i.c.v.) administration of a GLP‐1R antagonist reduced the ability of restraint stress to activate cFos expression by A2 neurons that co‐express prolactin‐releasing peptide (PrRP) (Maniscalco, Zheng, Gordon, & Rinaman, 2015). Although central GLP‐1R antagonism likely alters neural responses to stress across many brain regions, one potential explanation is that PrRP+ A2 neurons express GLP‐1R and receive axonal input from GLP‐1+ neurons in rats, which would be consistent with reports that a subset of catecholaminergic NTS neurons receive close appositions from the axons of PPG neurons and express GLP‐1R in mice (Cork et al., 2015; Llewellyn‐Smith et al., 2013; Richard et al., 2015).

Unlike catecholaminergic cNTS neurons, PPG neurons in mice do not express mRNA for GLP‐1R (as determined by single‐cell reverse transcription polymerase chain reaction (RT‐PCR)), and whole‐cell patch recording data indicate that PPG neurons do not respond to GLP‐1R agonists (Hisadome, Reimann, Gribble, & Trapp, 2010). However, as GLP‐1 neurons are glutamatergic (Zheng, Stornetta, et al., 2014), a lack of GLP‐1R expression does not preclude the possibility that GLP‐1 neurons are coupled via glutamatergic synapses. There is no published evidence regarding the presence or absence of GLP‐1R mRNA expression by identified GLP‐1 or noradrenergic/PrRP neurons in rats, and the ultrastructural features of GLP‐1 neurons and their synaptic associations within the cNTS has not been reported in either rats or mice.

This study used light and confocal microscopy to examine potential anatomical interactions (1) between GLP‐1+ and PrRP+ A2 neurons within the cNTS of adult male rats, and (2) between GLP‐1+ profiles in rats and between PPG/YFP+ profiles in mice. Using electron microscopy, we further examined the ultrastructural features of GLP‐1+ profiles within the rat cNTS. After synaptic contacts between GLP‐1+ profiles were confirmed, we used RNAscope to examine whether cNTS noradrenergic/PrRP+, GABAergic, and/or GLP‐1+ neurons express mRNA for GLP‐1R in rats. Finally, we revisited the possibility that PPG neurons in mice express functional GLP‐1R. For this purpose, Ca^2+^ imaging was performed in ex vivo slices from Glu‐Cre/Rosa26‐GCaMP3 mice to assess both somatic and dendritic responses of PPG neurons to GLP‐1, and a new transgenic mouse crossbreeding strategy was used to investigate potential colocalization of fluorescent reporter genes identifying PPG‐ and GLP‐1R‐expressing neurons.

## 2 MATERIALS AND METHODS

### 2.1 Animals

#### 2.1.1 Rats

Adult male Sprague–Dawley rats weighing 250–350 g were used for the present report. Rats were pair‐housed in a temperature‐controlled (22–25 °C) colony room with a 12:12 hr light–dark cycle (lights on at 0700). Food and water were available ad libitum. All experimental procedures using rats were conducted at the University of Pittsburgh or at Florida State University, conformed to NIH regulations detailed in the *Guide for the Care and Use of Laboratory Animals*, and were approved by the relevant Institutional Animal Care and Use Committees.

#### 2.1.2 Mice

Three transgenic strains of mice were used: (1) PPG‐Cre/GCaMP3 mice expressing Cre recombinase (Cre) and the Ca^2+^ indicator GCaMP3 selectively in PPG/GLP‐1 neurons, as previously reported (Holt, Llewellyn‐Smith, Reimann, Gribble, & Trapp, 2017); (2) PPG‐YFP mice expressing YFP selectively in PPG/GLP‐1 neurons (Hisadome et al., 2010); (3) GLP‐1R‐Cre/tdRFP mice expressing tdRFP selectively in GLP‐1R‐bearing cells (Cork et al., 2015). For this study, PPG‐YFP mice were crossed with GLP‐1R‐Cre/tdRFP mice to obtain mice with simultaneous expression of two different fluorescent reporter genes, one identifying PPG/GLP‐1 neurons, and the other identifying GLP‐1R‐expressing neurons. Crosses were genotyped as described previously (citations above) for the individual transgenes, and only mice positive for all three transcripts (i.e., Cre, YFP, and tdRFP) were used in the immunofluorescence study.

### 2.2 Immunocytochemical reagents

Glucagon‐like peptide‐1 was localized in rat tissue sections using a rabbit polyclonal antiserum and a mouse monoclonal antibody. The rabbit polyclonal (T‐4363, Peninsula Laboratories, San Carlos, CA; RRID: AB_518978) was generated against a synthetic peptide corresponding to amino acids 7–37 of the human GLP‐1 sequence. Specificity of this antiserum as applied to rat and human brain tissue has been documented (Zheng et al., 2014), including an absence of immunolabeling after pre‐absorbing the primary antiserum overnight at 4 °C with a 10‐fold higher concentration of synthetic GLP‐1 (7–37) acetate salt (Bachem, Torrance, CA, USA, H‐9560) before tissue incubation. The mouse monoclonal antibody was generated against N‐terminal residues 7–19 of the human GLP1 peptide, and was provided by Dr. Guibao Gu (RRID: AB_2314562). Specificity of this antibody for GLP‐1 has been documented (Gu et al., 2013), including (in our laboratory) an absence of immunolabeling after pre‐absorbing the primary antiserum overnight at 4 °C with a 10‐fold higher concentration of synthetic GLP‐1 (7–37) acetate salt (Bachem, H‐9560) before tissue incubation. Its selectivity also was tested against other cleavage products of the PPG gene, and was reported to weakly immunolabel GLP‐2, which is expressed along with GLP‐1 in the same neurons (Gu et al., 2013). PrRP was localized using a rabbit polyclonal antiserum (H‐008‐52, Phoenix Pharmaceuticals, Inc., Burlingame, CA, USA) raised against the synthetic 31 amino acid sequence of rat PrRP peptide. Antibody specificity has been documented by the manufacturer and also within our laboratory, based on positive and specific immunolabeling of a subset of dopamine beta hydroxylase‐positive noradrenergic neurons in the rat caudal brainstem (Maniscalco et al., 2015; Maniscalco, Kreisler, & Rinaman, 2013), and fully consistent with the reported distribution of PrRP mRNA expression and immunolabeling in mice and rats (Ellacott, Lawrence, Rothwell, & Luckman, 2002; Lawrence, Celsi, Brennand, & Luckman, 2000). The chicken anti‐GFP antibody (Catalogue #AB13970, lot #623923; Abcam, Cambridge, MA; RRID: AB_300798) used to localize YFP in mice was raised against a recombinant full length protein corresponding to green fluorescent protein (GFP). This antibody yields a single band on western blot (manufacturer's technical information), and specificity was demonstrated in this study by a lack of immunolabeling in tissue sections from wildtype rats and mice. Affinity purified secondary antibodies conjugated to fluorescent markers, biotin, and horseradish peroxidase (HRP) were purchased from Jackson ImmunoResearch Laboratories (West Grove, PA). ExtrAvidin‐HRP (Catalogue #E‐2886) was purchased from Sigma‐Aldrich (St. Louis, MO).

### 2.3 Tissue preparation for microscopy

#### 2.3.1 Rats

Rats were deeply anesthetized by i.p. injection of a lethal dose of sodium pentobarbital (Fatal Plus, 100 mg/kg; Butler Schein, Columbus, OH) and then perfused transcardially with fixative, as detailed below.

For light microscopic immunocytochemical localizations, rats (*n* = 4) were perfused with 300 mL of 0.1 M sodium phosphate‐buffered saline (PBS) containing 4% paraformaldehyde (PF; Sigma‐Aldrich, St. Louis, MO), 1.37% lysine and 0.22% sodium‐meta‐periodate (McLean & Nakane, 1974). Fixed brains were removed from the skull, postfixed for 24 hr, and then transferred to 20% sucrose solution for at least 24 hr. Brains were blocked and then sectioned serially at 35 μm using a freezing stage sliding microtome (Leica SM2000 R; Leica Biosystems, Wetzlar, Germany). Sections were collected sequentially in six vials of cryopreservant solution (Watson, Wiegand, Clough, & Hoffman, 1986) such that each vial contained sections at a frequency of 210 μm throughout the caudal brainstem. Vials of tissue were stored at −20 °C prior to immunocytochemical processing.

For transmission electron microscopic (TEM) analyses, one rat was perfused with 150 mL of 1.5% acrolein (Polysciences, Inc., Warrington, PA) and 2.0% PF in PBS followed by 100 mL of 2.0% PF in the same buffer. A second rat was perfused with 300 mL of a mixture of 2% PF and 0.5% TEM grade glutaraldehyde (Sigma‐Aldrich) in 0.2 M sodium cacodylate buffer. Each brain was removed from the skull, blocked to separate the forebrain from the brainstem, and postfixed in 2.0% buffered PF for 6 hr at 4 °C.

Rats used for RNAscope in situ hybridization (*n* = 4) were perfused with 100 mL physiological saline solution followed by 300 mL ice‐cold 4% PF in 0.1 M sodium phosphate buffer (PB). Brains were removed from the skull, postfixed in the same fixative overnight at 4 °C, incubated in 20% sucrose solution for 16–18 hr, and cut into six series of 25‐μm thick sections using a freezing stage sliding microtome. Tissue sections were collected in cryopreservant solution and stored at −20 °C until use.

#### 2.3.2 Mice

Adult mice (*n* = 5; three male, two female) generated by crossing PPG‐YFP mice with GLP‐1R‐Cre/tdRFP mice were transcardially perfused with heparinized ice‐cold PBS followed by 4% PF. Fixed brains were removed from the skull, postfixed in 4% PF overnight, and cryoprotected in 30% sucrose. Coronal brainstem sections (30 μm) were cut using a cryostat (Bright Instruments, Luton, Bedfordshire, UK).

### 2.4 Light microscopic immunocytochemical localizations

#### 2.4.1 Immunoperoxidase labeling of rat tissue

Cryopreserved sections were brought to room temperature and washed in multiple changes of 10 mM sodium PB on a rocker table. One vial of tissue sections from each brain was processed for immunoperoxidase localization of GLP‐1 using the rabbit polyclonal GLP‐1 antiserum. Sections were pretreated by incubation in 1.0% sodium borohydride followed by 0.15% hydrogen peroxide to increase antibody penetration and reduce nonspecific background. After PB rinsing, sections were then incubated in a 1:10 K dilution of the primary antibody in PB containing 0.3% Triton‐X and 1% normal donkey serum for 24–48 hr at 4 °C. Following multiple washes in PB the tissue was transferred to a 1:200 dilution of biotin‐conjugated affinity purified donkey anti‐rabbit secondary antibody and incubated for 2 hr at room temperature with agitation. Thereafter the sections were subjected to multiple PB washes prior to immunoperoxidase localization of GLP‐1 using Vectastain reagents (Vector Laboratories, Burlingame, CA) and diaminobenzidine (DAB) catalyzed with hydrogen peroxide.

#### 2.4.2 Immunofluorescent labeling of rat tissue

Dual immunofluorescence localization of GLP‐1 and PrRP in rat tissue sections was achieved using the mouse monoclonal GLP‐1 antibody and the rabbit PrRP polyclonal antiserum. Sections were pretreated by incubation in 1.0% sodium borohydride to increase antibody penetration and reduce nonspecific background. Following PB washes the tissue was incubated in mouse anti‐GLP‐1 overnight at room temperature, followed by multiple washes in PB prior to and following a 3‐hr room temperature incubation in affinity purified donkey anti‐mouse secondary antibody conjugated to HRP. Sections were then incubated in Tyramide conjugated to CY5 or FITC for 10 min at room temperature. After GLP‐1 labeling, sections were treated with 0.5% H2O2 for 15 min to remove residual peroxidase activity, followed by three 10‐min rinses with PB. The immunofluorescence processing was then repeated using the rabbit polyclonal PrRP antiserum at a dilution of 1:5 K, affinity purified donkey anti‐rabbit secondary antibody conjugated to HRP, and tyramide conjugated to CY3. Sections were mounted on gelatin‐coated slides and coverslipped using Vectashield Hard Set Mounting Medium (Vector Laboratories).

#### 2.4.3 Immunoperoxidase labeling of mouse tissue

Yellow fluorescent protein immunoperoxidase‐labeled brainstem sections from six male and two female PPG‐YFP mice were generated in previously published projects that focused on other research questions (Llewellyn‐Smith et al., 2013; Llewellyn‐Smith, Reimann, Gribble, & Trapp, 2011). Thus, while no new immunoperoxidase‐labeled mouse material was generated for this study, the cNTS‐focused microscopic analysis of this material has not been previously reported. Close appositions by PPG‐YFP axons onto PPG‐YFP dendrites were identified in sections labeled using chicken anti‐GFP (Abcam; 1:20 K; AB_13970) and a Ni‐intensified DAB reaction, as previously reported (Llewellyn‐Smith et al., 2011, 2013). Close appositions by PPG‐YFP axons onto PPG‐YFP cell bodies were identified in sections labeled using the same chicken anti‐GFP antibody diluted 1:400 K, followed by an imidazole‐intensified DAB reaction.

#### 2.4.4 Immunofluorescent labeling of mouse tissue

Yellow fluorescent protein immunoreactivity was detected using chicken anti‐GFP (1:500; Abcam). tdRFP expression was detected with an antibody raised against dsRed‐Express (1:500; #632496, Takara Bio, Mountain View, CA, USA). Free‐floating sections were incubated for 48 hr at 4 °C with primary antibodies in blocking solution (PBS with 0.3% triton X‐100, 1% BSA, 1% Normal Goat Serum, 1% Normal Donkey Serum) followed by incubation with Alexa Fluor 488‐conjugated goat anti‐chicken antibody (1:1,000; #A‐11039, ThermoFisher Scientific) and/or Alexa Fluor 568‐conjugated donkey anti‐rabbit antibody (1:1,000; #A‐10042, ThermoFisher Scientific) in blocking solution for 2 hr. Sections were subsequently mounted and coverslipped for microscopic analysis of fluorescent labeling.

### 2.5 Electron microscopic immunoperoxidase localizations

The fixed caudal brainstem from each rat perfused for ultrastructural analysis was sectioned at 100 μm using a Vibratome 1000 Plus (MyNeuroLab, Richmond, IL). Sections were collected sequentially in two wells of PBS such that each well contained sections at a frequency of 200 μm. Both wells of tissue were rinsed in PB for 30 min prior to and following a 10 min incubation in 1.0% sodium borohydride. The sections were then incubated in 1.0% hydrogen peroxide for 10 min, washed in PB for 30 min, transferred to cryoprotectant (Watson et al., 1986) and placed in a −80 °C freezer for 1 hr. Sections were returned to room temperature over 30 min, washed in 0.1 M Tris‐buffered saline (TBS) for 30 min, and then treated with a blocking solution containing 1.0% bovine serum albumin, 3.0% normal donkey serum, and 0.4% Triton X‐100 in TBS for 30 min. One well of sections was incubated overnight at room temperature with agitation in the GLP‐1 rabbit polyclonal antiserum (1:7 K) and the other well in the GLP‐1 mouse monoclonal antibody (1:15 K), both diluted with the blocking serum. Sections were subsequently washed in TBS for 30 min prior to and following a 1‐hr incubation in species‐specific biotinylated secondary antibodies at a dilution of 1:250. Vectastain reagents (Vector Laboratories) and DAB catalyzed with hydrogen peroxide were used to generate the immunoperoxidase reaction product.

Following the DAB reaction the sections were washed in multiple changes of PB and prepared for plastic resin infiltration and embedding. This involved postfixation of sections in a 1.0% osmium tetroxide solution (Electron Microscopy Sciences, Hatfield, PA) containing 1.5% potassium ferricyanide (Sigma‐Aldrich) for 30 min followed by multiple PB washes to clear unbound osmium from the tissue. Sections were then dehydrated using 15‐min changes in increasing concentrations of ethanol (50, 50, 70, 70, 95, 95, 100, 100%) and passed through four 15‐min changes of acetone. The last acetone wash was followed by sequential hour‐long incubations of sections in 50, 70, and 95% Epon‐Araldite plastic resin diluted with acetone followed by three consecutive hour‐long incubations in 100% resin. Each section was then flat embedded in 100% resin between acrylic sheets, followed by resin polymerization in a 60 °C oven.

### 2.6 RNAscope in situ hybridization

#### 2.6.1 Co‐localization of mRNA for GLP1R and PPG or GAD1

Selected tissue sections through the caudal medulla of each rat were removed from cryopreservant solution and washed for 1 hr in four changes of 50 mM TBS (pH 7.4). GLP1R, PPG, and GAD1 mRNAs were identified using Rn‐Glp1r probe (315221, Accession No. NM_012728.1, Target Region: 292‐1166), Rn‐Gcg probe (315471‐C2, Accession No. NM_012707.2, Target Region: 2‐1006), and Rn‐GAD1 probe (316401‐C2, Accession No. NM_017007.1, Target Region: 950‐1872) probe, respectively, using RNAscope® Fluorescent Multiplex Assay (320850; ACDBio, San Francisco, CA). Sections were incubated in boiling (99–100 °C) 1× target retrieval solution (322001) for 7 min, washed in deionized H_2_O two times for 1 min each, immediately mounted onto Gold Seal™ UltraStick™ Adhesion Microscope Slides (3039‐02; ThermoFisher Scientific, Waltham, MA, USA), and air‐dried at room temperature for 2 hr. Slides were then dipped in 100% ethanol for 10 sec and air‐dried before creating a hydrophobic barrier around each tissue section using a Pap Pen (195506; ThermoFisher Scientific). The barrier was allowed to completely dry at room temperature overnight before proceeding to the next step. Unless otherwise noted, incubations were performed at 40 °C within a HybEZTM Oven, using the HybEZTM Humidity Control Tray. For each section, two to four drops of each of the following reagent solutions were used to cover tissue sections, followed by three 3‐min 1× washing buffer rinses at room temperature. Sections were incubated with Protease III (322337) for 30 min and washed in deionized H_2_O four times for 1 min each. Sections were then incubated with a cocktail of the two probes (Rn‐Glp1r plus either Rn‐Gcg‐C2 or Rn‐GAD1‐C2) for 2 hr, followed sequentially by amplification steps with Amp1 for 30 min, Amp2 for 15 min, Apm3 for 30 min, and Amp4‐AltC (#332857) for 15 min. Sections were incubated with Amp5 for 30 s at room temperature and immediately covered with Fluoromount‐G® (0100‐01; Southern Biotech, Birmingham, AL, USA) mounting medium, without rinsing.

#### 2.6.2 Co‐localization of GLP1R mRNA and PrRP immunolabeling

GLP1R mRNA was first identified using the Rn‐Glp1r probe (315221) with RNAscope® 2.5 HD Detection Reagent—BROWN (322,310; ACDBio). For this purpose, sections through the cNTS were removed from cryopreservant solution and washed 1 hr in four changes of PB. Sections were treated 30 min in H_2_O_2_ pretreatment solution (322335) at room temperature, washed 30 min with four changes of PB, rinsed 1 min in deionized water, and mounted onto Gold Seal UltraStick Adhesion Microscope Slides (ThermoFisher Scientific, Waltham, MA, USA). Conditions for incubations and wash were similar as described above for dual RNAscope, unless otherwise noted. After drying overnight at room temperature, sections were incubated with Protease IV (322336) for 18 min at room temperature and washed in 10.0 mM TBS three times at 2 min each. Sections were then incubated with Rn‐Glp1r probe for 2 hr, followed sequentially by amplification steps Amp1–Amp6 according to the manufactuler's protocol. The last step (Amp7) was modified to convert the DAB immunoperoxidase staining into a fluorescent labeling. After Amp6 and washing, sections were incubated 10 min at room temperature in Cy3‐conjugated TSA plus solution (tyramine signal amplification plus, NEL744E001KT; PerkinElmer Waltham, MA, USA) at 1:450 dilution with TSA plus amplification diluent, followed by four changes of PB washing over 30 min. After completion of RNAscope labeling, sections were incubated in rabbit anti‐PrRP antiserum (1:3 K) for ~18 hr at room temperature, washed in PB, and incubated in Alexa 647‐conjugated donkey anti‐rabbit secondary antibody (1:600) for 2 hr. After PB rinsing, sections were allowed to dry, and then dehydrated/defatted and coverslipped using Cyroseal60 (VWR, Radnor, PA, USA).

### 2.7 Microscopy data collection and analysis

#### 2.7.1 Light microscopy in rat tissue

The distribution and density of GLP‐1+ cells and processes within the cNTS was characterized in rats using both immunoperoxidase‐ and immunofluorescence‐labeled material. An Olympus BX51 photomicroscope equipped with a Hamamatsu camera (Hamamatsu Photonics, Hamamatsu, Japan) was used to collect brightfield and epifluorescence images, and a Leica TCS SP8 confocal microscope was used to collect optical slice images and *z*‐stacks. Leica LAS 4.0 image collection software was used to generate maximum intensity *z*‐projections and 3D maximum intensity projections (MIPs), and the 3D visualization module was used for rendering/reconstructing tissue volumes. Region of interests (ROIs) from each 3D MIP were extracted to reveal details of associations between fluorescently labeled profiles.

#### 2.7.2 Light microscopy in mouse tissue

An Olympus BH‐2 brightfield microscope was used to examine immunoperoxidase‐labeled sections revealing YFP+ neurons and processes. Close appositions between YFP+ axon terminals and YFP+ cell bodies and dendrites were identified using a 100× oil immersion objective. YFP+ terminals lying adjacent to YFP+ somata or dendrites were classed as closely apposed when there was no space visible between the profiles. In Ni‐DAB‐reacted tissue sections, the dense black reaction product prevented detection of appositions in which YFP+ axons overlay YFP+ cell bodies. This problem was overcome by further diluting the anti‐GFP antibody to 1:400,000, and using a lighter brown immunoperoxidase reaction product to detect YFP+ profiles. In these sections, YFP+ terminals were considered to closely appose an underlying YFP+ cell body when the terminal and the cell body were in the same focal plane. Digital images of appositions were collected as TIFF files using a SPOT Insight Model 18.2 firewire color camera running SPOT RT software v5.2 (Diagnostic Instruments, Inc., Sterling Heights, MI). Adobe PhotoShop was used to adjust image sharpness, brightness, and contrast, and to create montages.

Immunofluorescence was visualized on an upright widefield microscope (Leica Wetzlar, Germany) equipped with GFP (Chroma 49002 Chroma Technology, Bellows Falls, Vermont, USA) and red fluorescent protein (RFP) (Chroma 49008) filter sets. Images were captured using a color camera (Retiga 3000; QImaging Surrey, BC, Canada) and Q‐Capture Pro 7 software (QImaging).

#### 2.7.3 Electron microscopy in rat tissue

Figure 5 illustrates the approach used for the collection of ultrastructural data from rat tissue samples. Flat‐embedded 100 μm vibratome sections were transilluminated and photographed using an Olympus SZX10 Research Stereomicroscope to identify the location of immunopositive neurons within the cNTS (Figure 5a). Sections were then trimmed to a trapezoid that included immunopositive neurons (Figure 5a), the trapezoid was glued to a blank resin stub, and the sample was sectioned using a diamond knife (Diatome, Hatfield, PA) and ultramicrotome (Reichert Wien, Austria). As soon as tissue was encountered, series of ultrathin (~600 Å) sections bracketed by 0.35 μm thick sections were collected sequentially from each sample. Thick sections were collected on microscope slides, and intervening ultrathin sections were collected on formvar coated narrow slot grids (Figure 5b,c). One of the two thick sections between each ultrathin series remained unstained (Figure 5e), while the second was stained with toluidine blue (Figure 5d,f). Alternate series of thick and ultrathin sections were sequentially collected (Figure 5c) until immunopositive neurons were no longer visible. As immunoperoxidase reaction product is differentially concentrated in the top 10–15 μm of vibratome sections (due to limited penetration of immunocytochemical reagents), this approach ensured that all labeled profiles would be identified in the ultrastructural analysis. It also allowed individual profiles to be identified in multiple ultrathin sections with those closest to the surface being densely labeled (often obscuring the ultrastructural features of labeled profiles), and those deeper in the block more discretely labeled (and thus, more revealing of ultrastructural features).

Unstained thick sections were examined to unambiguously identify labeled neurons (Figure 5e) while staining of adjacent thick sections with toluidine blue permitted identification of the same profiles in relation to histological landmarks (e.g., bundles of myelinated fibers; Figure 5f). Through careful registration of these landmarks with ultrathin sections in the electron microscope it was possible to precisely identify the same labeled profiles in both thick (Figures 5b,e) and ultrathin (Figure 5g) sections. Grids were not counterstained with either uranyl acetate or lead citrate; contrast was enhanced only with insertion of objective apertures into the beam path in the microscope column. This enhanced our ability to identify the electron density of GLP‐1 immunoperoxidase reaction product.

Each ultrathin section was systematically sampled using a previously described approach (see Figure 4 in Rinaman, Card, Schwaber, & Miselis (1989)). This involved serial horizontal passages across the full extent of the ultrathin section, taking note of landmarks and immunopositive profiles identified in the thick sections. GLP‐1+ profiles were photographed at both low and high magnification in one or more ultrathin sections. Digital electron micrographs of immunopositive cells were captured to document the subcellular distribution of immunoperoxidase reaction product. Immunopositive axon terminals were examined to determine the distribution of immunoperoxidase reaction product, the cellular elements that they contacted and, when visible, the symmetry of synaptic contacts.

#### 2.7.4 RNAscope imaging

Low magnification images were acquired with a KEYENCE epifluorescent microscope (BZ‐X700). Confocal images were acquired using a Leica TCS SP8 confocal microscope, 100× oil objective and 3× digital zoom. The Atto550 fluorophore (Rn‐Glp1r) was excited using a 552 nm OPSL laser, and Atto647 (Rn‐Gcg or Rn‐GAD1) was excited using a 638 nm Diode laser Images were collected sequentially using Leica LAS 4.0 image collection software, and using the 3D Visualization module for rendering/reconstructing tissue volumes. A total of 21 0.28 μm‐thick optical sections were collected to generate a maximum intensity *z*‐projection and a 3D MIP. A ROI from the 3D MIP was optically sectioned and extracted to reveal details of the labeling profiles.

### 2.8 Calcium imaging in ex vivo slices in mouse

Adult Glu‐Cre/Rosa26‐GCaMP3 mice (*n* = 3; one female, two males; 10–15 weeks old) were anesthetized with isoflurane and decapitated. The brainstem was quickly removed from the skull and placed in ice‐cold high‐Mg^2+^/low‐Ca^2+^ artificial cerebrospinal fluid (ACSF; composition in millimolar: 2.5 KCl, 200 sucrose, 28 NaHCO_3_, 1.25 NaH_2_PO_4_, 7 Glucose, 7 MgCl_2_, 0.5 CaCl_2_; pH 7.4). A total of 200 μm‐thick coronal brainstem slices were cut on a vibratome (7000smz2; Campden Instruments, Loughborough, UK) and incubated in recovery solution (in millimolar: 3 KCl, 118 NaCl, 25 NaHCO_3_, 1.2 NaH_2_PO_4_, 2.5 Glucose, 7 MgCl_2_, 0.5 CaCl_2_; pH 7.4) at 34 °C for 45 min. Sections were then transferred to standard ACSF (in millimolar: 3 KCl, 118 NaCl, 25 NaHCO_3_, 10 Glucose, 1 MgCl_2_, 2 CaCl_2_; pH 7.4) and left at 34 °C for a minimum of 30 min before imaging. All solutions were constantly bubbled with 95% O_2_/5% CO_2_.

Imaging was performed using a Zeiss Axioskop 2FS widefield microscope (Zeiss, Oberkochen, Germany) with a 40× water immersion lens. Slices were continuously superfused with 32 °C standard ACSF at a flow rate of 3–4 mL/min. Recombinant human GLP‐1 (7–37) was obtained from Peprotech (London, UK) and glutamate from Sigma‐Aldrich (Gillingham, Dorset, UK). Stock solutions were made up in H_2_O and diluted at least 1,000× when added to the ACSF. GCaMP3 was excited using an LED light source (CoolLED pE300white; QImaging) at 460 nm (±25 nm) for 250 ms every 5 s. Images were captured at 12‐bit on a charge‐coupled device camera (Q‐Click; QImaging). Recordings were imported into FIJI image analysis software. XY‐drift was adjusted for using the StackReg plugin. ROIs and an area for determining background fluorescent intensity were outlined and the mean pixel intensity calculated for each ROI. Background intensity was subtracted from each ROI and recordings were adjusted for bleaching using a cubic polynomial function. Fluorescence intensity data are presented as Δ*F*/*F*_0_ with *F*_0_ being the average fluorescence intensity 5 min prior to the first stimulus and Δ*F* being the fluorescence intensity, *F*, minus *F*_0_. “*N*” numbers indicate number of analyzed ROIs. Responses were quantified by calculating area under the curve for more than 5 min during the stimulus, starting at the beginning of the stimulus. Because they were found not to be normally distributed, summary data are presented as box plots with whiskers, with the median indicated and whiskers marking the 10th and 90th percentile, respectively. Statistical significance of differences was determined using nonparametric statistical testing as described in the legend of Figure 11.

## 3 RESULTS

### 3.1 Light microscopy

**Figure 1:**
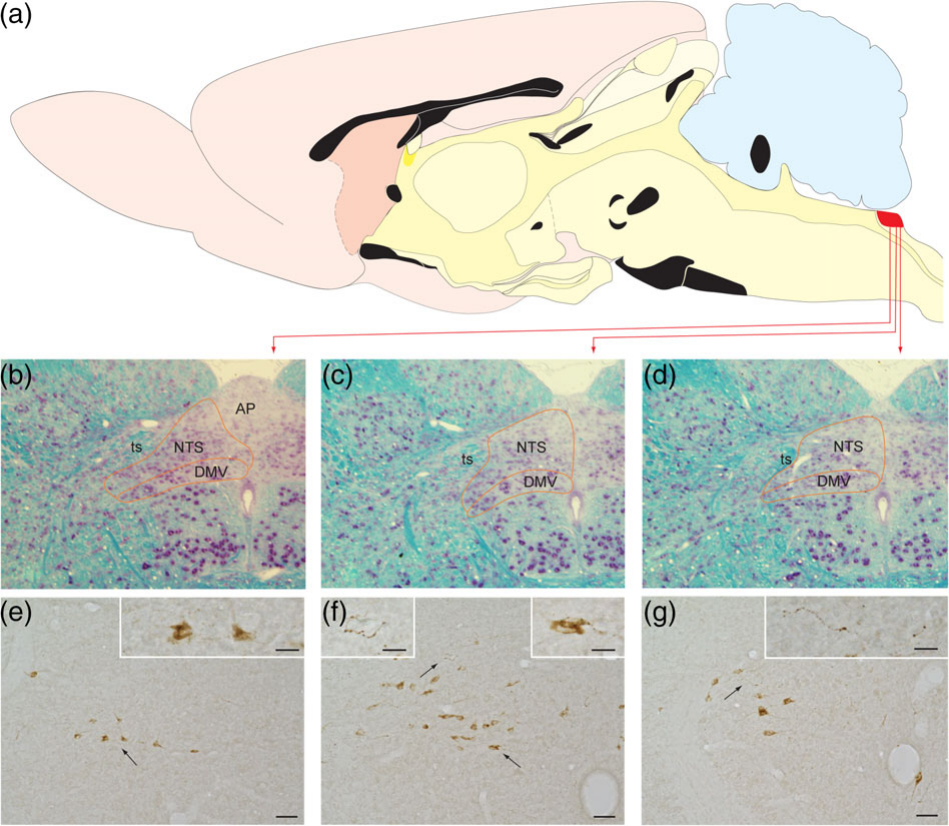
The portion of the caudal nucleus of the solitary tract (cNTS) within which GLP‐1 neurons are sequestered is illustrated for the rat brain. (a) The location of the cNTS within the hindbrain dorsal motor vagal complex is marked in red. (b–d) The cytoarchitecture of this cNTS region is depicted in three coronal sections stained with the Kluver–Barrera method. (e–g) Immunoperoxidase labeling reveals GLP‐1+ profiles in coronal sections through rostrocaudal levels comparable to those shown in panels (b–d). Arrows indicate specific regions shown at higher magnification in the panel insets. Scale bars: 50 μm in (e–g), and 20 μm for the insets within those panels. AP = area postrema; DMV = dorsal motor nucleus of the vagus; NTS = nucleus of the solitary tract; ts = solitary tract [Color figure can be viewed at http://wileyonlinelibrary.com]

**Figure 2:**
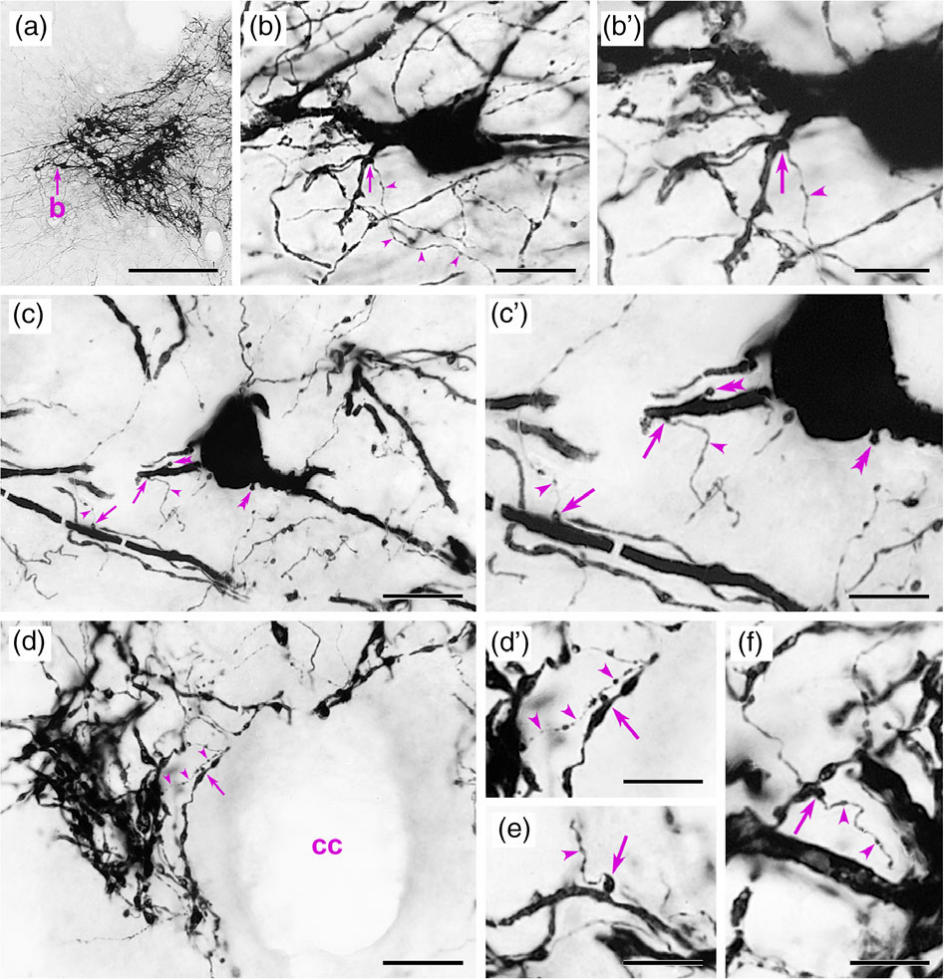
Immunoperoxidase localization of yellow fluorescent protein (YFP) immunoperoxidase labeling within the caudal medulla of pre‐proglucagon (PPG)‐YFP mice, in which YFP+ axons frequently form close appositions on YFP+ dendrites. In each panel, arrowheads indicate the YFP+ axonal source of YFP+ terminals that form close appositions. (a, b, b′) In the caudal nucleus of the solitary tract (a), a YFP+ terminal forms a close apposition (arrow in b, b′) on the proximal dendrite of a PPG‐YFP neuron. (c, c′) A YFP+ terminal closely apposes (right arrow) a rostral PPG‐YFP neuron that has spines (double arrowheads) on its soma and a proximal dendrite. Another YFP+ terminal forms a close apposition (left arrow) on a nearby YFP+ dendrite. (d, d′) Near the cc, a YFP+ dendrite receives a close apposition (arrow) from a YFP+ terminal. (e, f) Other close appositions (arrows) from YFP+ terminals onto YFP+ dendrites. Scale bars: 250 μm in (a); 20 μm in (b–d); 10 μm in (b′, c′, d′, e, f). cc = central canal [Color figure can be viewed at http://wileyonlinelibrary.com]

Light microscopic analyses confirmed prior findings regarding the caudal brainstem distribution of GLP‐1+ neurons in rats, and of YFP+ neurons in PPG‐YFP mice. In both species, GLP‐1+ or YFP+ neurons were present within the portion of the NTS caudal to the area postrema (cNTS; see Figure 1 for rat GLP‐1+ profiles, and Figure 2 for mouse YFP+ profiles), with additional GLP‐1+/YFP+ neurons forming an arc extending from the ventrolateral cNTS into the lateral brainstem tegmentum at the same rostrocaudal levels. In both rodent species, a rich plexus of varicose GLP‐1+/YFP+ axons arborized within the cNTS, coextensive with GLP‐1+/YFP+ cell bodies and dendrites. The latter were more readily visible as YFP+ profiles in immunoperoxidase‐labeled material from PPG‐YFP mice (Figure 2), in which the distribution of YFP+ neurons was as previously reported (Llewellyn‐Smith et al., 2011; Llewellyn‐Smith et al., 2013). In mice, most YFP+ somata occupied the cNTS, with additional YFP+ cell bodies located in the intermediate reticular nucleus (IRT) and on the midline. Analysis of close appositions between YFP+ axons, cell bodies, and dendrites was restricted to the cNTS. Each of the eight mice examined displayed clear examples of YFP+ axon terminals closely apposed to YFP+ dendrites (Figure 2). Convincing examples of YFP+ terminals apposing YFP+ cell bodies also were detectable in sections that were lightly stained for immunoperoxidase using dilute anti‐GFP and a brown reaction product. In these sections, varicose YFP+ axons could be followed as they travelled over, under or alongside YFP+ somata (not shown).

**Figure 3:**
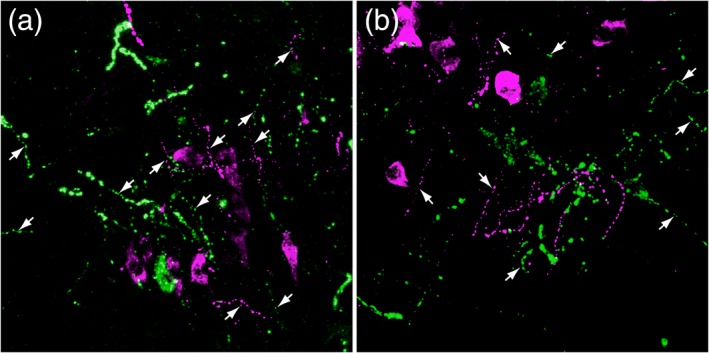
Epifluorescent dual localization of GLP‐1+ (green) and PrRP+ (magenta) neurons and processes in two different fields (a, b) within the rat caudal nucleus of the solitary tract, demonstrating the overlapping distribution of these separate neural populations. Both neural populations give rise to thick dendritic processes and thin varicose axons (arrows) [Color figure can be viewed at http://wileyonlinelibrary.com]

**Figure 4:**
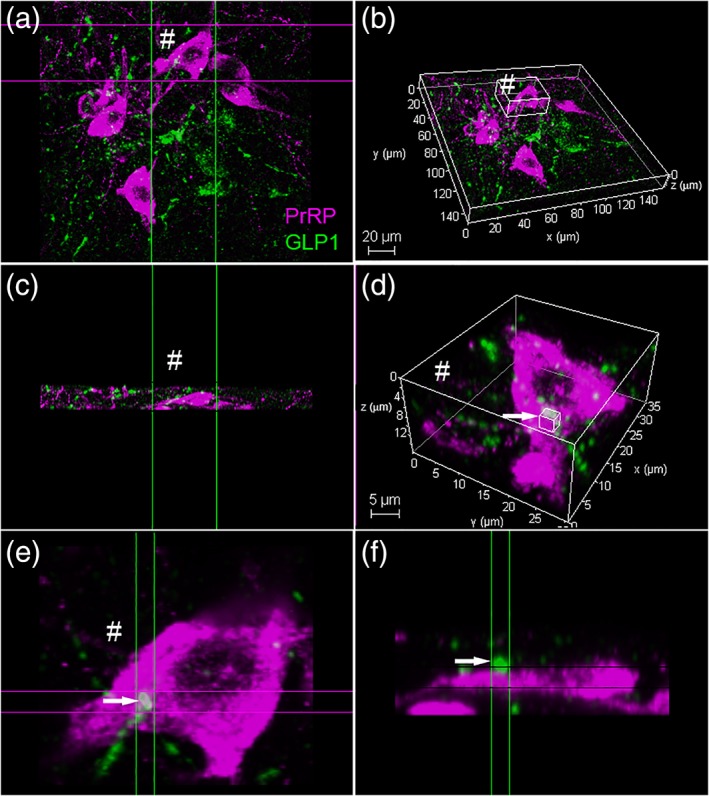
Confocal imaging of dual PrRP (magenta) and GLP‐1 (green) immunofluorescence in the rat cNTS. (a) Maximum projection (*z*‐stack) 3D image, slightly rotated in (b) to reveal the same region. (c) A “side view” image stack of the same PrRP+ neuron indicated by # in panels (a and b). (d) Higher‐magnification view of the same # neuron, revealing close appositions formed by GLP‐1+ terminals (green) onto the PrRP neuron. The smaller boxed region (arrow) is depicted at even higher magnification in (e) (“top down” view) and (f) (“side view”), revealing no apparent gap between the green and magenta profiles (arrow). Scale bars: (b) 20 μm; (d) 5 μm. GLP‐1 = glucagon‐like peptide‐1; PrRP = prolactin‐releasing peptide [Color figure can be viewed at http://wileyonlinelibrary.com]

In rat tissue samples, dual immunofluorescent labeling confirmed the generally overlapping but noncolocalized distribution of GLP‐1+ and PrRP+ positive neurons within the cNTS (Figure 3). GLP‐1+ and PrRP+ neurons typically gave rise to thick dendrites that tapered and branched as they extended from their parent cell body, and networks of thin varicose GLP‐1+ axons (arrows in Figure 3) arborized in areas coextensive with GLP‐1+ and PrRP+ neurons. Confocal microscopy was used to investigate close appositions between GLP‐1+ and PrRP+ profiles at higher magnification in optical images collected using a 100× oil objective (Figure 4). Maximum projection and rotation of 3D images confirmed the presence of close appositions between GLP‐1+ axonal varicosities and the cell bodies of PrRP+ noradrenergic neurons (Figure 4d–f).

### 3.2 Ultrastructural findings

**Figure 5:**
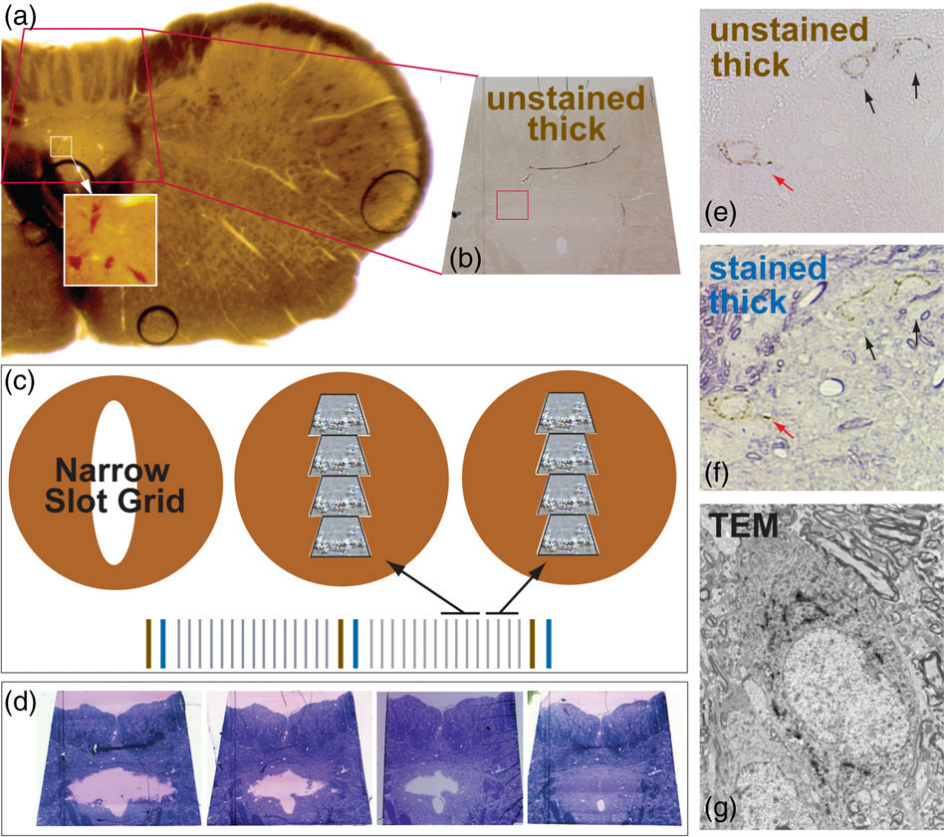
The strategy used for electron microscopic analysis of GLP‐1‐labeled profiles within the rat caudal nucleus of the solitary tract (cNTS) is illustrated. (a) Plastic‐embedded 100 μm‐thick vibratome section through the cNTS which was labeled for GLP‐1 immunoperoxidase before embedding. Transillumination reveals the location of GLP‐1+ neurons (white boxed area); this area was subsequently trimmed (red trapezoid in (a), same region depicted in (b)). (c) An ultramicrotome was used to generate an alternating series of two thick (0.35 μm) followed by 15 ultrathin (600 Å) sections. One thick section between each ultrathin series was left unstained (b, e) and the other thick section was stained with toluidine blue (d, f). This systematic approach optimized identification of GLP‐1+ profiles in all regions of the cNTS, permitting identification of the same profiles in thick and ultrathin sections. For example, panel (g) shows the ultrastructure of the same cell identified at the light microscopic level (red arrows in panels (e) and (f)). TEM = transmission electron microscopy [Color figure can be viewed at http://wileyonlinelibrary.com]

**Figure 6:**
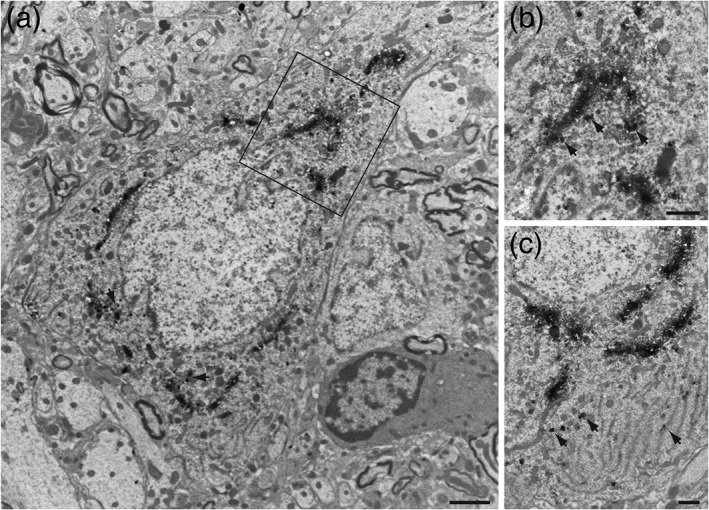
Electron micrographs illustrating the cellular localization of GLP‐1 immunoperoxidase reaction within the rat caudal nucleus of the solitary tract. (a) GLP‐1+ neurons had a long axis of 10–15 μm and a short axis of 7–10 μm with a prominent centrally placed cell nucleus. (b) Immunoperoxidase labeling within neurons was densely concentrated in Golgi complexes surrounding the cell nucleus and in large vesicles associated with the cis face of the Golgi (arrows in (b)) or distributed among cisternae of the rough endoplasmic reticulum in the cell cytoplasm (arrows in (c)). Scale bars: 2 μm in each panel

**Figure 7:**
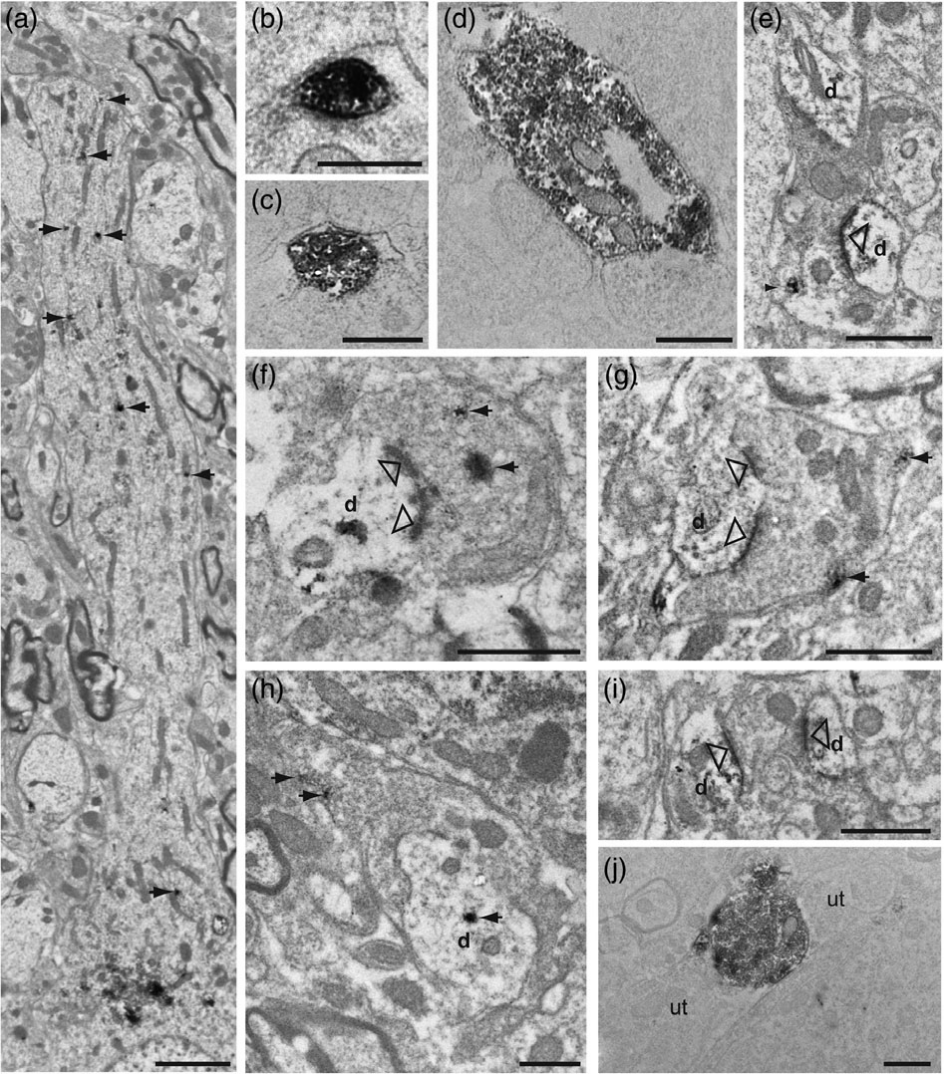
Electron micrographs illustrating GLP‐1+ processes in the rat caudal nucleus of the solitary tract. (a) Immunopositive vesicles derived from the Golgi complexes of the cell soma extended into the dendritic arbor to produce discrete labeling (arrows). (b, c) Immunopositive axon terminals displayed a range of labeling density that was directly correlated with the proximity of the ultrathin section to the surface of the flat embedded tissue. Profiles within the top few microns of the tissue (b) contained dense concentrations of immunoperoxidase reaction product that filled the profile, while those deeper in the tissue (c) displayed more punctate labeling. GLP‐1+ terminals were densely filled with 40 nm lucent spherical vesicles and occasional dense core vesicles. Immunopositive terminals formed appositions with both unlabeled (b–d) and labeled (e–h) dendrites. (f, g) When synapses (open arrowheads) were present within the plane of section they were asymmetric in character and, in many cases, formed multiple synapses with the same profile. Immunopositive dendrites also were recipient of synaptic input from immunonegative terminals that exhibited both asymmetric/excitatory (i) and symmetric/inhibitory characteristics. (j) GLP‐1+ axon varicosities often were closely apposed to unlabeled axon terminals (ut) filled with 40 nm lucent spherical vesicles. Scale bars: (a) 2 μm; (b) 500 nm; (c, f) 600 nm; (d) 800 nm; (g–i) 1 μm. d, dendrite; ut, unlabeled terminal

Electron microscopy was used to investigate the ultrastructural features of GLP‐1+ profiles in the rat cNTS, including the apparent close appositions between GLP‐1+ profiles. In electron micrographs, GLP‐1+ profiles included oval neurons with a long axis of 10–15 μm, a short axis of 7–10 μm, and an organelle‐filled cytoplasm that was typically concentrated at one pole of the long axis (Figure 5g, Figure 6a). The cell's centrally placed nucleus exhibited dispersed chromatin, and characteristically exhibited shallow invaginations. The cytoplasm contained numerous Golgi cisternae distributed around the cell nucleus, prominent cisternae of rough endoplasmic reticulum arranged individually and in stacks, and large numbers of mitochondria. Prominent primary dendrites extended from the polar aspects of the cell (Figures 6a and 7a), within which mitochondria and large vesicles were interspersed among linearly arranged microtubules (Figure 7a).

Light micrographs of unstained and stained thick sections revealed immunoperoxidase reaction product concentrated in dense aggregates surrounding the cell nucleus and extending into the proximal portion of primary dendrites (Figure 5e,f). In electron micrographs of the same cells (Figure 5g and 6a), the peroxidase reaction product was concentrated within the Golgi complexes surrounding the cell nucleus and in the 100–120 nm vesicles associated with cisternae on the cis face of Golgi complexes (arrows, Figure 6b), in the cytoplasm among cisternae of RER (arrows, Figure 6c), and within dendrites (Figure 7a). Within the neuropil, the density and distribution of peroxidase reaction product correlated directly with the proximity of the ultrathin section to the surface of the vibratome section. Labeled axons near the surface of the section displayed dense concentrations of peroxidase that filled the entire profile (Figure 7b,c). Immunolabeling in axons was progressively more discretely localized in sections taken at increasing depths from the surface of the vibratome section. At intermediate depths (3–6 μm), flocculent peroxidase reaction accumulated on the external surface of 40 nm spherical vesicle membranes and mitochondria, and labeling filled the labeled profile (Figure 7d). In deeper sections (7–12 μm) labeling became increasingly punctate, and was often differentially associated with collections of lucent spherical 40 nm vesicles (Figure 7e,f) or with individual 100–120 nm vesicles (Figure 7h). The pattern of dendritic labeling was consistent, with electron‐dense immunoperoxidase being discretely localized irrespective of proximity to the surface of the vibratome section. Typically, peroxidase labeling within dendrites was confined to 100–120 nm vesicles (Figure 7a,f) or presented as flocculent aggregates on the membranes of microtubules and mitochondria (Figure 7e–i).

GLP‐1+ axon terminals were filled with lucent spherical 40 nm vesicles and occasional large dense core vesicles (Figure 7b–h, j), and formed synaptic contacts with both labeled (i.e., GLP‐1+; Figure 7e–g) and unlabeled dendrites (not illustrated). GLP‐1+ terminals also were observed closely apposed to unlabeled axon varicosities containing lucent spherical 40 nm vesicles (Figure 7j). Consistent with light microscopic observations in both mice (Figure 2) and rats (Figure 3), axo‐dendritic synaptic contacts between GLP‐1+ profiles were prevalent within the vicinity of GLP‐1+ cell bodies. Presynaptic GLP‐1+ terminals typically displayed aggregates of 40 nm lucent spherical vesicles adjacent to the presynaptic membrane, and a prominent postsynaptic density (i.e., asymmetric, Gray's Type 1 synapses; Figure 7e–g). The majority of such contacts terminated on intermediate and distal dendrites (i.e., diameter ≤1 μm) and, in many cases, a single GLP‐1+ axon terminal formed multiple synapses with a single GLP‐1+ dendrite (Figure 7f,g). GLP‐1+ dendrites were also contacted by unlabeled axon terminals containing either lucent spherical 40 nm vesicles (Figure 7i) or containing pleomorphic 60 nm vesicles (not illustrated).

### 3.3 RNAscope results

**Figure 8:**
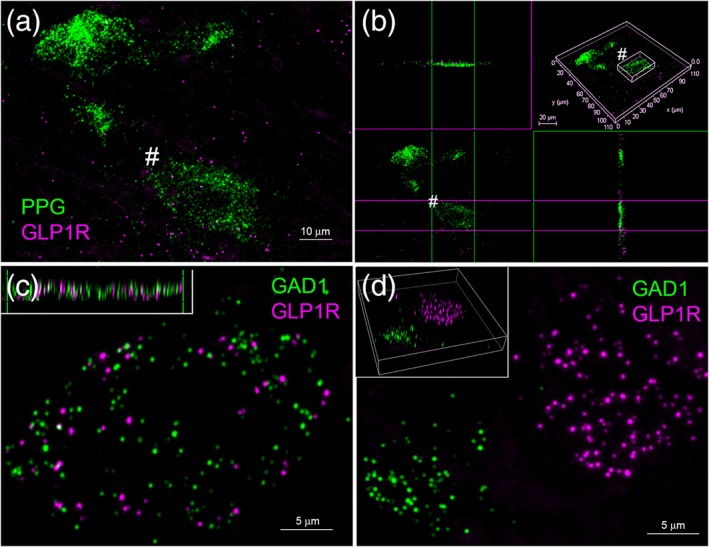
Confocal imaging of dual RNAscope fluorescent in situ hybridization labeling in rat caudal nucleus of the solitary tract (cNTS). (a) Maximum projection *z*‐stack showing PPG mRNA (green) and GLP1R mRNA (magenta) transcripts. (b) Rotated image stack from the same area to depict views from both sides of one PPG mRNA‐expressing neuron (marked with # in panel (a)), revealing magenta GLP1R mRNA transcripts that lie adjacent to (but not within) the PPG‐expressing neuron. (c) Maximum projection *z*‐stack showing GAD1 mRNA (green) and GLP1R mRNA (magenta) transcripts, with the rotated 3D side view (inset) demonstrating intracellular colocalization of both transcripts. (d) Maximum projection *z*‐stack showing GAD1 mRNA (green) and GLP1R mRNA (magenta) transcripts in a different field within the cNTS, demonstrating lack of colocalization of the two transcripts. Scale bars: (a) 10 μm; (c, d) 5 μm. GLP‐1R = GLP‐1 receptor, PPG = pre‐proglucagon [Color figure can be viewed at http://wileyonlinelibrary.com]

**Figure 9:**
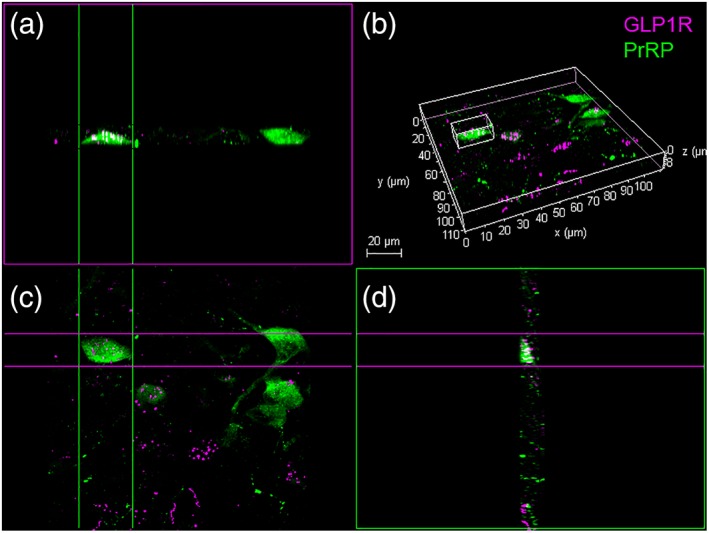
Confocal imaging of PrRP immunolabeling (green) and GLP1R mRNA (magenta) within the rat caudal nucleus of the solitary tract. (a) “Side view” of a rotated image stack depicting a double‐labeled neuron. The same neuron is shown from a “top view” in a maximum projection *z*‐stack image in panel (c), and from an “end‐on” image stack view in panel (d). Within the same field of view (visible in each panel), other double‐labeled PrRP+ neurons are located nearby, although some lack intracellular colocalization of GLP1R mRNA (e.g., the neuron located on the right in panels (a) and (c)). Scale bar: (b) 20 μm. GLP‐1R = GLP‐1 receptor; PrRP = prolactin‐releasing peptide [Color figure can be viewed at http://wileyonlinelibrary.com]

Dual in situ hybridization using RNAscope revealed both PPG and GLP‐1R mRNA transcripts within the rat cNTS and medullary reticular formation (Figure 8). GLP1R mRNA transcripts also were present in additional regions of the caudal medulla, including the area postrema, inferior olive, midline raphe nuclei, and around the central canal. Within the cNTS, GLP1R mRNA transcripts were sometimes present within the vicinity of PPG mRNA transcripts, but generally were not located in close proximity. In a few cases, however, scattered GLP1R mRNA transcripts appeared to overlap with PPG mRNA transcripts (Figure 8a). In those cases, closer inspection using high‐magnification confocal microscopy and rotation of 3D projection images failed to demonstrate intracellular colocalization of GLP‐1R and PPG mRNA transcripts (Figure 8b). Conversely, GLP1R mRNA was localized within some GABAergic neurons (i.e., expressing GAD1 mRNA) within the cNTS and adjacent reticular formation (Figure 8c), whereas other GABAergic neurons did not co‐express GLP1R mRNA (Figure 8d). When RNAscope localization of GLP‐1R mRNA was combined with immunofluorescent localization of PrRP, confocal microscopy revealed that GLP‐1R mRNA transcripts were co‐localized within a subset of PrRP+ noradrenergic A2 neurons (Figure 9).

### 3.4 Transgenic mouse cross‐breeding strategy

**Figure 10:**
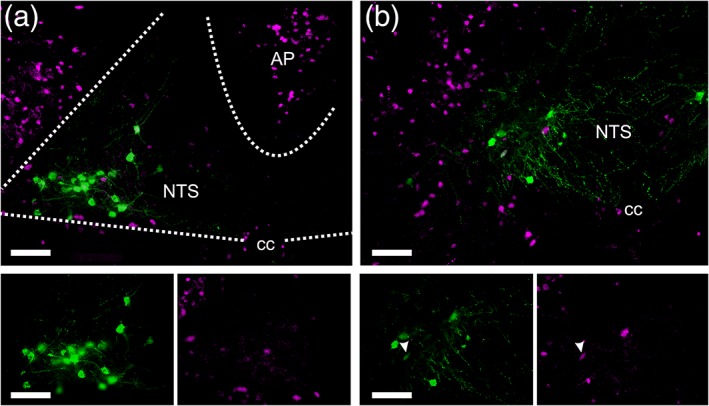
Photomicrographs showing yellow fluorescent protein (YFP)‐positive pre‐proglucagon (PPG) neurons (green) and tdRFP‐positive GLP‐1R neurons (magenta) in coronal NTS sections from transgenic mice cross‐bred to display both reporters. (a) At Bregma −7.6 mm, most PPG neurons are located in the lateral part of the NTS. Some GLP‐1R neurons are observed in the NTS, but a higher density is seen in the AP. The lower panel shows the PPG neurons and GLP‐1R neurons separately to confirm lack of colocalization of YFP and tdRFP in the same cells. (b) At Bregma −8.0 mm, NTS PPG neurons are located more medially than at more rostral levels. This section shows a rare case of colocalization of YFP and tdRFP in the same cell (white arrowheads in the magnified view panels located below (b)), suggesting that this lone PPG neuron expresses GLP‐1R. Scale bars = 100 μm. AP = area postrema; NTS = nucleus of the solitary tract; cc = central canal [Color figure can be viewed at http://wileyonlinelibrary.com]

Pre‐proglucagon (PPG)‐YFP mice were crossed with GLP‐1R‐Cre/tdRFP mice to obtain mice that expressed the YFP reporter in PPG neurons, and the tdRFP reporter in GLP‐1R‐expressing cells. Both tdRFP and YFP are expressed as soluble proteins, and labeled cell bodies were detected by immunofluorescence using antibodies raised against dsRed (to label RFP) and against GFP (to label YFP), as illustrated in Figure 10. In five mice (three male, two female), the incidence of co‐localization of tdRFP in YFP+ cells was quantified within the cNTS, IRT, and midline raphe ventral to the hypoglossal nucleus. Of 736 NTS PPG neurons, only seven were RFP+. Similarly, only 1 out of 322 IRT PPG neurons and 2 out of 65 midline PPG neurons were RFP+. Thus, the proportion of mouse PPG neurons that were double‐labeled for GLP‐1R fluorescent reporter was very small (i.e., 0.95% in the NTS, 0.31% in the IRT, and 3.08% along the midline).

### 3.5 Ca^2+^ imaging

**Figure 11:**
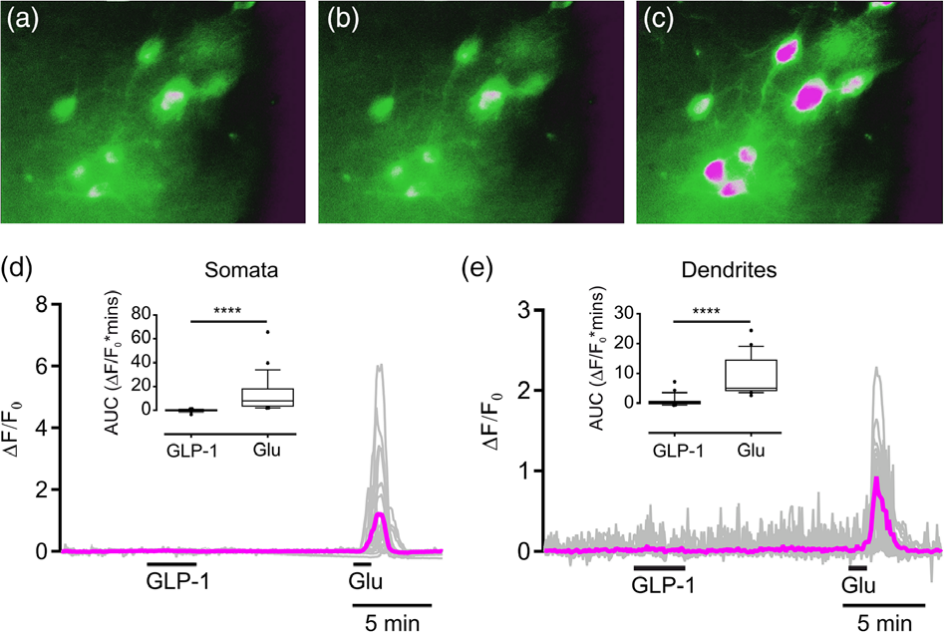
Optical recordings of the intracellular Ca^2+^ concentration in pre‐proglucagon (PPG) neurons reveal a lack of response to GLP‐1. (a–c) Pseudocolored micrographs showing Ca^2+^ levels in eight PPG neurons under different conditions: control (a), 100 nM GLP‐1 (b), 100 μM glutamate (c). (d) The gray traces show the individual somatic responses of 27 PPG neurons (from three mice) to 100 nM GLP‐1 and 0.1 mM glutamate, and the magenta trace shows the mean response from these cells. (e) Gray traces show the individual responses of 23 PPG dendrites (from three mice) to 100 nM GLP‐1 and 0.1 mM glutamate, and the magenta trace shows the mean response from these dendrites. In both (d) and (e), statistical analysis of the responses expressed as AUC is shown as a box plot with whiskers, with the median indicated and whiskers marking the 10th and 90th percentile, and outliers represented by black filled circles. Significance was determined with a Wilcoxson test. ****p < .0001. AUC = area under the curve; GLP‐1 = glucagon‐like peptide‐1 [Color figure can be viewed at http://wileyonlinelibrary.com]

The response of identified PPG neurons to bath application of GLP‐1 and glutamate was investigated in ex vivo slices through the caudal medulla in Glu‐Cre/Rosa26‐GCaMP3 mice (*n* = 3 mice; Figure 11). When slices were exposed to 100 nM GLP‐1 for 3 min, none of the identified PPG neurons (0 out of 27) exhibited an intracellular Ca^2+^ response, whereas all 27 neurons responded to 100 μM glutamate (Figure 11d). This was also the case for the readily visible dendrites of PPG neurons; no intracellular Ca^2+^ response was observed on application of GLP‐1, but all sampled dendrites responded to 100 μM glutamate (*n* = 23 dendrites; Figure 11e). Thus, bath application of GLP‐1 did not elicit a functional response in either the somatic or dendritic compartments of PPG neurons.

## 4 DISCUSSION

In the initial phase of this study, we demonstrated that GLP‐1+ varicose axons within the rat cNTS form close appositions with neural profiles that are immunopositive for PrRP, and also with GLP‐1+ profiles. Subsequent analysis of immunoperoxidase‐labeled material from PPG‐YFP mice, in which YFP reporter gene expression is driven by the PPG promoter, revealed that YFP+ axonal varicosities form close appositions with YFP+ cell bodies and dendritic profiles within the mouse cNTS, consistent with our observations in rats. Electron microscopy confirmed that appositions between axonal and dendritic GLP‐1+ profiles in rats include synaptic contacts, revealing a local synaptic network among hindbrain GLP‐1 neurons. Guided by these anatomical observations, we set out to determine whether PrRP+ and/or GLP‐1+ neurons express GLP‐1R in either rats or mice, using molecular (RNAscope in situ hybridization) and physiological (Ca^2+^ imaging) approaches. Based on the results obtained, we speculate that GLP‐1/PPG neurons release glutamate, GLP‐1, and perhaps other signaling molecules within a local cNTS neural network to modulate the activity of noradrenergic PrRP neurons, GABA neurons, and other GLP‐1/PPG neurons. However, as GLP‐1/PPG neurons do not express GLP‐1R in either rats or mice, synaptic coupling among these neurons likely uses glutamate or other signaling molecules rather than GLP‐1.

*GLP‐1+ axonal appositions with PrRP+ noradrenergic neurons*. Our light‐microscopic observations of presumptive contacts between GLP‐1+ axons and PrRP+ dendrites in rats are consistent with published reports using transgenic mice. In those reports, YFP+ fibers originating from PPG‐expressing (i.e., GLP‐1) neurons were observed to form close appositions with tyrosine hydroxylase‐positive catecholaminergic neurons within the cNTS (Llewellyn‐Smith et al., 2013; Richard et al., 2015). Our confocal observations in rats extend those anatomical results to another rodent species, and further demonstrate that catecholaminergic cNTS neurons targeted by GLP‐1+ inputs include A2 noradrenergic neurons that express PrRP. Further, we report that at least a subset of these PrRP+ neurons express mRNA for GLP‐1R, demonstrating their potential to be modulated by GLP‐1 signaling.

Hindbrain GLP‐1 neurons and PrRP+ A2 neurons are robustly activated in rats after exposure to visceral or cognitive stressors, and published evidence supports the view that both neural populations contribute to the central control of stress responsiveness and motivated behavior via widespread axonal projections that reach multiple CNS regions implicated in these processes (Banihashemi & Rinaman, 2006; Bechtold & Luckman, 2006; Ellacott et al., 2002; Gu et al., 2013; Holt & Trapp, 2016; Kreisler, Davis, & Rinaman, 2014; Larsen et al., 1997; Lawrence et al., 2000; Lawrence, Ellacott, & Luckman, 2002; Lawrence, Liu, Stock, & Luckman, 2004; Llewellyn‐Smith et al., 2013; Maniscalco et al., 2013, 2015; Maniscalco & Rinaman, 2018; Myers & Rinaman, 2002; Rinaman, 1999b, 2003, 2010; Trapp & Cork, 2015; Zheng & Rinaman, 2013)**.** GLP‐1R expression by PrRP+ neurons could contribute to local modulatory effects on Ca^2+^ signaling within the local somatodendritic compartment of PrRP neurons, and/or distal axo‐axonic interactions that affect release of norepinephrine and PrRP within CNS target regions that receive axonal input from both cNTS neural populations (Maniscalco & Rinaman, 2017; Maniscalco et al., 2013; Rinaman, 2010). Interestingly, restraint stress activates cFos expression by both GLP‐1+ and PrRP+ neurons in rats, identifying both neural populations as “stress‐sensitive” (Maniscalco et al., 2015). However, the ability of restraint stress to activate cFos expression in PrRP+ neurons and also within the anterior bed nucleus of the stria terminalis (which receives axonal input from PrRP+ and GLP‐1+ neurons) is significantly attenuated in rats after central (i.c.v.) administration of Exendin‐9, a specific GLP‐1R antagonist (Maniscalco et al., 2015). Our new anatomical evidence suggesting that GLP‐1+ fibers provide direct input to PrRP+ neurons, coupled with RNAscope in situ hybridization evidence that PrRP neurons express GLP‐1R mRNA, support the view that PrRP neurons are directly responsive to GLP‐1, and that GLP‐1R signaling at least partially underlies their stress sensitivity. Dual‐labeling electron microscopy will be necessary to confirm the synaptic nature of close appositions between GLP‐1+ and PrRP+ neurons in both mice and rats, although earlier work suggests that at least half of the close appositions observed at the light microscopic level represent actual synaptic contacts (Pilowsky, Llewellyn‐Smith, Lipski, & Chalmers, 1992). However, unlike the point‐to‐point release of glutamate and GABA, which appears to require synaptic contacts, release of GLP‐1 and other neuropeptides is likely not similarly restricted (van den Pol, 2012). Local volumetric diffusion of GLP‐1 to GLP‐1R's expressed by nearby cNTS cells, including PrRP and GABA neurons, could facilitate peptidergic modulatory control over these neurons, even in the absence of synaptic contacts.

*GLP‐1+ synaptic inputs to GLP‐1+ profiles*. Using single immunoperoxidase labeling and electron microscopy, we document the existence of another local synaptic circuit within the cNTS, in which GLP‐1+ neurons form axo‐dendritic synaptic contacts with GLP‐1+ target neurons. These presumptive contacts were readily visible at the light‐microscopic level in both rats and mice, and their synaptic nature was confirmed at the ultrastructural level in rats. The asymmetric (i.e., excitatory, Gray's type 1 (Peters, Palay, & Webster, 1991)) features of these GLP‐1‐to‐GLP‐1 synaptic contacts within the caudal NTS is consistent with evidence that GLP‐1 neurons are glutamatergic, and provide asymmetric synaptic inputs to neural targets within the rat hypothalamus (Sarkar et al., 2003; Zheng et al., 2014).

The presence or absence of synaptic contacts between GLP‐1 neurons in mice has not been previously reported. Instead, in an earlier electrophysiological study using perforated patch recordings in ex vivo slices from PPG‐YFP mice, no GLP‐1R‐mediated effects were observed on the resting membrane potential, membrane resistance, or firing frequency of identified PPG neurons and no GLP‐1R mRNA was detected in single cell RT‐PCR analysis (Hisadome et al., 2010). Given our new anatomical evidence for close appositions and synapses between PPG/GLP‐1 axon terminals and dendrites, we revisted the lack of physiological effects using a different approach. For this, ex vivo slices were prepared from mice in which the Ca^2+^ indicator GCaMP3 was selectively expressed in PPG neurons, followed by optical recordings of intracellular Ca^2+^ concentration in PPG cell bodies and dendrites before and after exposure to GLP‐1. This approach lacked the temporal resolution of electrophysiological recordings, but had higher spatial resolution and allowed analysis of several PPG neurons simultaneously. It also enabled us to specifically investigate the presence or absence of (distal) dendritic responses to GLP‐1 that do not propagate to the cell soma. Such responses would have gone undetected in our earlier electrophysiological study, but have been reported recently for 5‐HT acting on PPG neurons (Holt et al., 2017). The observed absence of GLP‐1‐induced changes in somatic or dendritic intracellular Ca^2+^ is consistent with the earlier electrophysiological reports and also with our inability to detect mRNA for GLP‐1 in those cells (Cork et al., 2015; Hisadome et al., 2010). Conversely, some catecholaminergic (i.e., TH+) cNTS neurons do express GLP‐1R in mice (Cork et al., 2015), consistent with new evidence (discussed below) that noradrenergic PrRP+ neurons in rats express mRNA for GLP‐1R.

*GLP‐1R is expressed by PrRP and GABA neurons, but not by GLP‐1 neurons*. RNAscope in situ hybridization was used to analyze PPG mRNA expression by phenotypically identified cells within the cNTS in rat tissue samples. For this purpose, some tissue sections were processed to visualize two different mRNA transcripts in the same section (i.e., PPG and GLP‐1R mRNAs, or GLP‐1R and GAD1 mRNAs), while other sections were processed to visualize GLP‐1R mRNA in combination with PrRP immunofluorescence. GLP‐1R mRNA transcripts were readily identified within subsets of GABAergic (GAD1 mRNA‐expressing) and PrRP+ neurons within the cNTS, but were never observed to colocalize within GLP‐1 (PPG mRNA‐expressing) neurons. Similarly, in transgenic mice that were bred to express two unique reporter genes to simultaneously but separately identify PPG‐ and GLP‐1R‐expressing neurons, only a very small number (<1%) of identified PPG neurons also expressed the GLP‐1R reporter gene.

We interpret these anatomical and physiological data as evidence that synaptic communication among GLP‐1 neurons in rats and mice does not use GLP‐1R‐mediated signaling. Further support for this argument comes from our earlier study investigating the effect of i.c.v. Exendin‐9 administration on stress‐induced recruitment of phenotypically identified cNTS neurons. As mentioned previously, Exendin‐9 attenuated the ability of restraint stress to activate cFos expression by PrRP+ neurons (Maniscalco et al., 2015), consistent with our new results indicating that PrRP neurons express mRNA for GLP‐1R. Conversely, i.c.v. Exendin‐9 did *not* attenuate stress‐induced activation of GLP‐1+ neurons within the cNTS of the same rats within that study (Maniscalco et al., 2015), consistent with the current report demonstrating a lack of GLP‐1R mRNA expression by GLP‐1 neurons in rats, or by PPG neurons in mice.

Glutamatergic and GLP‐1R signaling mechanisms interact in many brain regions (Gilman et al., 2003; Liu et al., 2017; Mietlicki‐Baase et al., 2013, 2014), and GLP‐1R signaling may modify excitatory synaptic transmission via cellular mechanisms involved in long‐term synaptic plasticity (Liu et al., 2017). Speculatively, as GLP‐1+ axonal varicosities in rats and YFP+ varicosities in transgenic PPG reporter mice are immunoreactive for vesicular glutamate transporter 2 (Trapp & Cork, 2015; Zheng et al., 2014), synaptic signaling between GLP‐1 neurons may be mediated by glutamate. Light‐evoked release of glutamate from PPG nerve terminals within the hypothalamic paraventricular nucleus has been demonstrated in mice (Liu et al., 2017); similarly, clonal intestinal L‐cells that secrete GLP‐1 express VGLUT2 and co‐release glutamate, hypothesized as important for intercellular signaling among intestinal L‐cells (Uehara et al., 2006). The relatively low density of GLP‐1+ fibers and terminals within the rat cNTS (see Figures 1 and 3) contrasts with the high density of GLP‐1+ terminals within the rat hypothalamus and limbic forebrain (Gu et al., 2013). However, YFP+ axons are abundant within the cNTS in PPG‐YFP mice (see Figure 2), whereas we have reported that GLP‐1 immunolabeling in the mouse cNTS produces limited axonal labeling, similar to rats (Lachey et al., 2005). Thus, it is possible that immunoreactive GLP‐1 neuropeptide is trafficked to the distal projections of GLP‐1/PPG neurons, with the local projections signaling primarily via glutamate. This remains to be determined using technical approaches with more sensitivity than immunolabeling. Given the lack of GLP‐1R expression by GLP‐1 neurons, GLP‐1 released either locally or distally is unlikely to directly alter subsequent neurotransmitter release from these neurons. Conversely, locally or distally released GLP‐1 may act at pre‐ or postsynaptic GLP‐1R receptors to modulate neuropeptide and/or glutamate or GABA signaling from subsets of A2 PrRP+ cells and GABA cells within the cNTS, given our new evidence that subsets of these neurons express GLP‐1R. Additional studies will be required to test these hypotheses, including studies to determine the axonal projections of PrRP+ neurons that either do or do not express GLP‐1R.

Quantitative results from retrograde neural tracing studies indicate that somewhere between 30 and 50% of GLP‐1/PPG neurons within the cNTS project to the ventral tegmental area, nucleus accumbens (Alhadeff, Rupprecht, & Hayes, 2012; Dossat, Lilly, Kay, & Williams, 2011), medial hypothalamus (Vrang, Hansen, Larsen, & Tang‐Christensen, 2007), paraventricular thalamic nucleus (Ong, Liu, Pang, & Grill, 2017), and/or spinal cord (Llewellyn‐Smith et al., 2015). Given the large number of CNS regions receiving axonal input from this relatively small population of hindbrain neurons (Gu et al., 2013), individual GLP‐1/PPG neurons likely have axon collaterals that branch to innervate more than one target site; indeed, such collateralization has been demonstrated for GLP‐1 neural inputs to the paraventricular and dorsomedial nuclei of the hypothalamus in rats (Vrang et al., 2007). If subsets of GLP‐1/PPG neurons innervate different central target sites, then local glutamatergic synaptic connections between these neurons within the cNTS might provide moment‐to‐moment coupling that coordinates the activities of different target‐specific populations. Alternatively, or in addition, synaptic coupling among GLP‐1/PPG neurons could provide a mechanism for simultaneous modulation or even “mass activation” of several projection‐specific populations.

In summary, multiple lines of evidence support the view that GLP‐1 neurons can influence the activity of local GABAergic and noradrenergic PrRP neurons via pathways that include GLP‐1R signaling, but may influence the activity of interconnected GLP‐1 neurons only via glutamatergic or other non‐GLP‐1R‐mediated mechanisms. The latter results represent new anatomical evidence for synaptic contacts by peptidergic neurons onto target neurons that do not express the cognate peptide receptor. While we are unaware of parallel results in other peptidergic neural systems, it would be surprising if this arrangement is unique to GLP‐1/PPG neurons. Additional studies are necessary to determine the neurochemical basis for GLP‐1/PPG interneuronal signaling, and the functional effects of such signaling on recruitment of GLP‐1 neurons and downstream target neurons within the CNS.
